# *DEF6* has potential to be a biomarker for cancer prognosis: A pan-cancer analysis

**DOI:** 10.3389/fonc.2022.1064376

**Published:** 2023-01-04

**Authors:** Ziming Yuan, Yuchen Zhong, Hanqing Hu, Weiyuan Zhang, Guiyu Wang

**Affiliations:** Cancer Center/Department of Colorectal Cancer Surgery, The Second Affiliated Hospital of Harbin Medical University, Harbin, Heilongjiang, China

**Keywords:** pan-cancer, DEF6, prognosis, tumor microenvironment, immune infiltration

## Abstract

**Introduction:**

*DEF6* is a gene associated with the immune system and is thought to play a crucial role in autoimmunity. There are few *DEF6*-related studies in cancer, and it is assumed that *DEF6* is a proto-oncogene. There is currently no pan-cancer analysis of *DEF6*, and we performed a systematic and comprehensive pan-cancer analysis of *DEF6* in an attempt to reveal its role and function in cancer.

**Methods:**

The data were analyzed by mining databases available to the public and by using R software. Moreover, immunohistochemistry was used to validate the results.

**Results:**

Our results revealed that *DEF6* is commonly aberrantly expressed in cancer and its expression is strongly correlated with survival prognosis in a variety of cancer types. Through correlation analysis we found that *DEF6* was associated with multiple immune genes and was closely related to immune infiltration. In the enrichment analysis, *DEF6* may have cross-talk with multiple cancer pathways and exert oncogenic or pro-cancer functions. In addition, we collected pathological samples from colorectal cancer patients for immunohistochemical analysis and found that patients with higher immunohistochemical scores had more lymph node metastases, higher CA199, and bigger tumor size.

**Discussion:**

Overall, *DEF6* expression is closely related to cancers and has the potential to act as a cancer biomarker.

## Introduction

Over 20,000 protein-coding genes have been identified, and the functions of most of them are not well understood (1). Pan-cancer analysis allows us to study the function of genes by *in silico* analysis. When a gene is not well understood in cancer, pan-cancer analysis can reveal differences and similarities in gene behavior across different cancers.

*DEF6* guanine nucleotide exchange factor (*DEF6*) is an immune-related gene that can promote T-cell receptor (TCR)-induced Ca^2+^ release and include activation of the transcription factor nuclear factor of activated T cells (NFAT) (2). Furthermore, *DEF6* is related to immunological synapse and antigen-stimulated T cells (3). *DEF6* can also regulate Th17 cell differentiation (4). In non-cancerous disease, *DEF6* deficiency can lead to reduced numbers of T and B cells, autoimmune diseases, hepatosplenomegaly, and bowel inflammation (5, 6). In malignant tumors, *DEF6* appears to be associated with a worse prognosis. In clear cell renal cell carcinoma, a high level of expression of *DEF6* predicts poor prognosis (7); in human osteosarcoma, a high level of expression of *DEF6* is associated with metastasis and poor prognosis (8); in ovarian carcinoma, a high level of expression of *DEF6* is associated with poor prognosis (9). Overall, a high level of expression of *DEF6* seems to be strongly associated with poor prognosis in patients, and studies in cancer are very limited. Based on the above studies, we hypothesize that *DEF6* is likely to be an oncogene. However, the details of the function of *DEF6* have not been fully revealed and have been validated in only a few tumors. A systematic pan-cancer analysis of *DEF6* would be valuable, and no pan-cancer analysis of *DEF6* is available. Therefore, we conducted a comprehensive, multi-omic, pan-cancer analysis for *DEF6*, using several public databases, with the intention of revealing the character of *DEF6* in cancers. The main objective of this study is to reveal the potential functions of *DEF6* in a variety of tumors through bioinformatics, to understand the oncological processes that *DEF6* may affect, and to validate the potential of *DEF6* as a diagnostic marker.

## Materials and methods

### Cancer data collection

A total of 33 types of cancer RNA-seq data were downloaded from the The Cancer Genome Atlas (TCGA) database. ENSG00000023892 (*DEF6*) was the target gene for data processing and extract. Normal tissue RNA-seq data were acquired from the Genotype-Tissue Expression (GTEx) database (https://commonfund.nih.gov/GTEx) (10). We transformed the RNA-seq data to TPM, and a log_2_(1+TPM) transformation was performed.

### Cell line data collection

A total of 1,406 cancer cell lines from 33 types of disease were downloaded from the Cancer Cell Line Encyclopedia (CCLE) database (https://sites.broadinstitute.org/ccle) (11, 12). The downloaded raw data were transformed into TPM and a log_2_(1+TPM) transformation was performed.

### Survival data collection

Curated clinical data (*n*=12,591), including high-quality survival outcomes (overall survival, progression-free interval, disease-specific survival, and disease-free interval), were extracted from a published paper (13).

### Analysis of RNA modification genes

Expression data for 44 marker genes for three classes of RNA modifiers [i.e., of N1-methyladenosine (m1A) (10), 5-methylcytosine (m5C) (13), and N6-methyladenosine (m6A) (14)] in each sample were extracted from downloaded RNA-seq data. In addition, Spearman correlation was used for the correlation between *DEF6* and RNA-modified genes.

### Analysis of immune-related genes

We extracted expression data for three classes of immune pathways [i.e., chemokine, major histocompatibility complex (MHC), and receptor] from the downloaded TCGA dataset and analyzed the Spearman’s correlation of *DEF6* expression with these genes.

### Tumor microenvironment and infiltration of immune cells

The “ESTIMATE” R package (v1.0.13) calculates stromal, immune, and ESTIMATE scores for each patient based on gene expression (15). We evaluated the scores of each patient’s immune cells using the Timer method *via* the “IOBR” R package (16).

### Tumor heterogeneity analysis

MuTect2 software processed the level 4 simple nucleotide variation dataset downloaded from TCGA. The tumor mutational burden (TMB) and mutant-allele tumor heterogeneity (MATH) for each tumor were calculated using the TMB and inner heterogeneity functions of the “maftools” R package (ver. 2.8.05), and the TMB and MATH scores were combined with gene expression data (17). We obtained tumor purity and microsatellite instability (MSI) data for each sample from previous studies and merged these with expression data, followed by Spearman’s correlation analysis (18, 19).

### Genetic alteration analysis

We analyzed genetic alterations using the cBio Cancer Genomics Portal (http://cbioportal.org), an open-access resource for interactive exploration of multidimensional cancer genomic datasets (20). In addition, the “Cancer Types Summary” submenu was used to analyze and visualize genetic alteration frequencies. To assess the relationship between DNA methylation and the copy number alteration (CNA) profile of *SUSd4*, the “mutation” module in the Gene Set Cancer Analyses (GSCA) (http://bioinfo.life.hust.edu.cn/GSCA/#/mutation) was utilized (21).

### Enrichment analysis

To construct the protein–protein interaction network (PPI), we used the STRING database (https://cn.string-db.org/) (14, 22–24). As for STRING parameters, the minimum interaction score was 0.15, and the top 50 relative proteins were obtained. The “clusterProfiler” (v4.4.4) R package was used to perform gene ontology (GO) and Kyoto Encyclopedia of Genes and Genomes (KEGG) pathway enrichment analysis.

For gene set enrichment analysis (GSEA), the GSEA software (v 3.0) was utilized, and samples were grouped by *DEF6* expression level (cut-off value 50%) (25). The KEGG symbol matrix was acquired from the Molecular Signatures Database (http://www.gsea-msigdb.org/gsea/downloads.jsp) to evaluate the potential pathways and mechanisms based on gene expression profiles and groupings, in which the minimum and maximum gene set function were set to 5 and 5,000 re-samplings, respectively (26).

### Patient information and immunohistochemistry

Tissue samples were harvested from colorectal cancer patients who underwent surgical treatment at the Department of Colorectal Surgery of the Second Hospital of Harbin Medical University, and a total 20 pairs of samples were obtained. Ethics approval for this study was obtained from the Second Affiliated Hospital of Harbin Medical University. The method of immunohistochemistry was consistent with previous studies (27, 28). We calculated the immunohistochemical (IHC) score as the ratio of positively stained cells to staining intensity. The IHC score can help us to assess the level of *DEF6* expression. The *DEF6* antibody was purchased from Abcam (ab279395). All procedures performed in this study were in accordance with the Declaration of Helsinki (1964) and its later amendments.

### Statistical analysis

Unpaired Wilcoxon tests were used to calculate the differential expression in normal and tumor samples. The Cox proportional hazards regression model was subsequently established using the coxph module of the “survival’” (v5.6-2) and “survminer”(v0.4.9) R packages to analyze the inner link between gene expression and prognosis. The “forestplot” R package (v2.0.1) was used to map the forest plot. We also calculated the optimal cut-off value of risk score using the “maxstat” R package (version 0.7-25), in which the minimum sample size was set to greater than 30%. We used the Spearman’s correlation method for correlation analysis and the Benjamin–Hochberg (BH) method for *p*-value adjustment.

Statistical analysis was carried out using R 4.2.0 software and the above-mentioned R packages.

## Results

### DEF6 expression analysis

**Figure 1:**
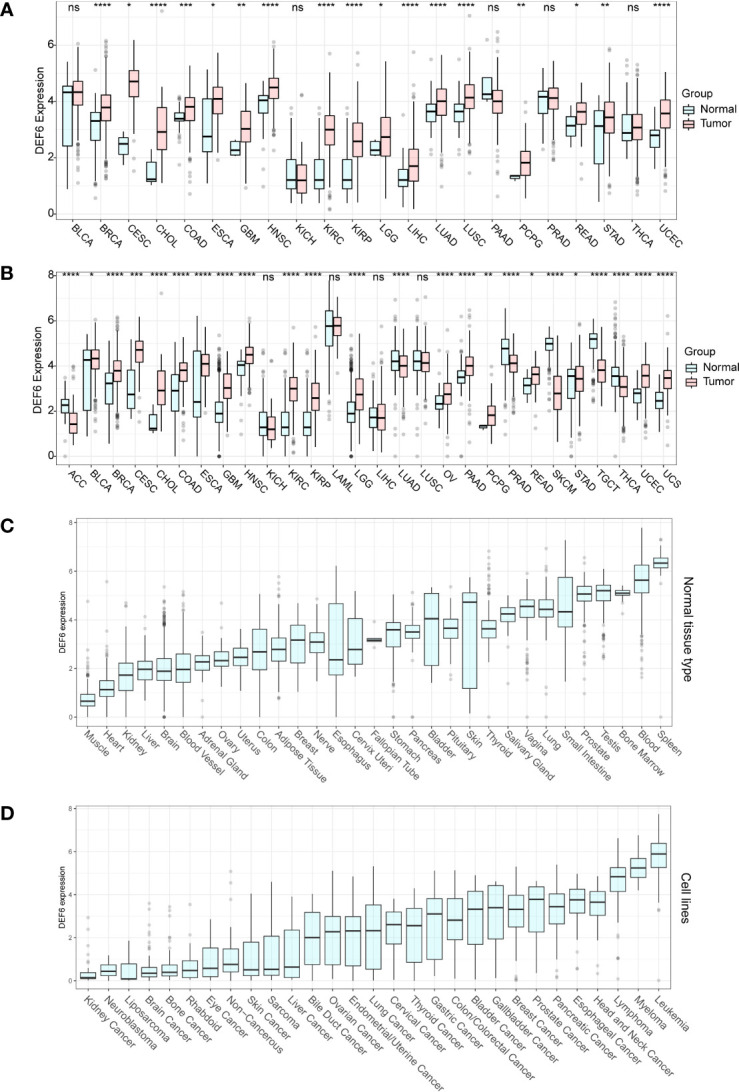
*DEF6* expression profile. **(A)** Differential expression of *DEF6* in TCGA dataset. **(B)** Differential expression of *DEF6* in TCGA dataset and the GTEx dataset. **(C)** Expression of *DEF6* in normal tissues. **(D)** *DEF6* expression at the cell line level. ns, not significant; *p < 0.05; **p < 0.01; ***p < 0.001; ****p < 0.0001.

High levels of expression of the gene in cancerous tissues imply a possible association with tumorigenesis or progression. We first analyzed the RNA expression of *DEF6* in cancer tissues compared with normal tissues using TCGA data (**Figure 1A**). However, the number of normal tissue RNA-seq data is insufficient; we extracted normal tissue data from the GTEx dataset to pair with TCGA cancers for comparability; as shown in **Figure 1B**, we found that *DEF6* was significantly aberrantly expressed in multiple cancers after supplemental normal tissue data. Next, we analyzed the expression of *DEF6* in normal tissues using the GTEx database, and concluded that *DEF6* showed a different distribution trend in normal tissues, as seen in **Figure 1C**, which shows that levels of *DEF6* expression were lower in muscle and heart tissues and higher in blood and spleen tissues. Finally, we analyzed trends in the levels of *DEF6* expression in cancer cell lines, which were lowest in kidney cancer, neuroblastoma, and liposarcoma cell lines and highest in leukemia, myeloma, and lymphoma cell lines (**Figure 1D**). Among solid tumors, the level of *DEF6* expression was highest in head and neck cancer, esophageal cancer, and pancreatic cancer cell lines.

### Survival analysis

**Figure 2:**
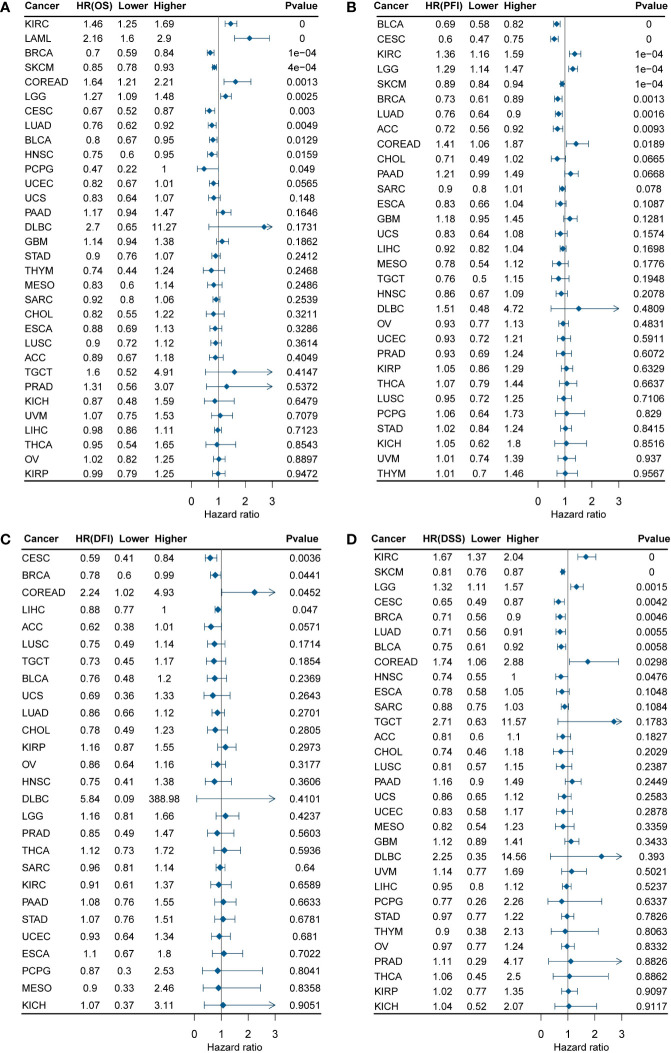
*DEF6* and survival situations. **(A-D)** Forest plots of *DEF6* expression and OS, PFI, DFI, and DSS. OS, overall survival; PFI, progression-free interval; DFI, disease-free interval; DSS, disease-specific survival; HR, hazard ratio.

To validate *DEF6* as a predictor of cancer prognosis, we calculated the relationship between *DEF6* expression and survival by univariate Cox survival analysis. **Figure 2**, a forest plot of *DEF6* mRNA expression versus overall survival (OS), shows the outcomes of progression-free interval (PFI), disease-free interval (DFI), and disease-specific survival (DSS) outcomes.

**Figure 3:**
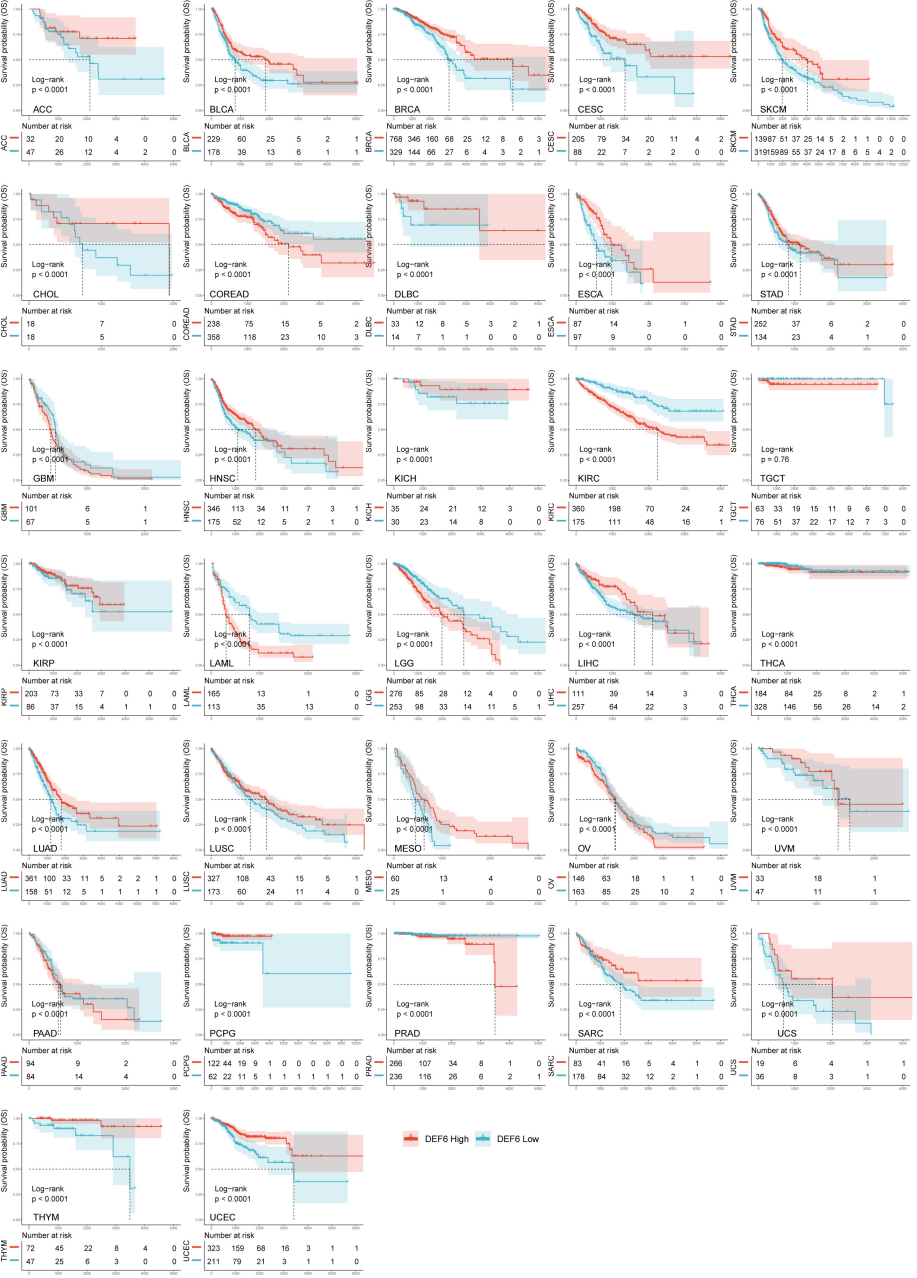
Analysis of the overall survival in multitumors with best cut-off method.

**Figure 2A** shows that a high level of *DEF6* mRNA expression is related to poor prognosis in KIRC, LAML, COREAD, and LGG and better prognosis in BRCA, SKCM, CESC, LUAD, BLCA, HNSC, and PCPG. As shown in **Figure 2B–D**, we found that a high level of *DEF6* expression in COREAD was associated with all four of the poorer prognostic outcomes, whereas *DEF6* expression in BRCA and CECS was associated with a better prognosis. This implies that *DEF6* may have different functions in different cancers, and that in COREAD *DEF6* may mainly play the role of oncogene. Next, we utilized algorithms to define an optimal cut-off point to group the samples and performed log-rank survival analysis (29). As shown in **Figure 3**, by using the best cut-off value method, it was possible to distinguish survival differences in 31 cancers but not in TGCT. A high level of expression of *DEF6* in ACC, BLCA, BRCA, CESC, SKCM, CHOL, DLBC, ESCA, STAD, HNSC, KICH, KIRP, LIHC, LUAD, LUSC, MESO, and UVM was associated with a better prognosis. High *DEF6* transcription levels were associated with a worse prognosis in COREAD, GBM, KIRC, LAML, LGG, THCA, OV, PAAD, and PRAD.

### Patient information and immunohistochemical analysis

We performed an IHC analysis to verify the relationship between *DEF6* and colorectal cancer. **Supplementary Figure 1A** shows an IHC colorectal cancer pathology section and B shows IHC results of paired normal tissues. We found significantly higher IHC scores for colorectal cancer tissue than for normal tissue (**Supplementary Figure 1C**). The IHC score of most cancerous tissues was 4 or 5, whereas the IHC score of most paired normal tissues was 2. We divided the patients into two groups—high and low level of expression—based on IHC scores. As shown in **Table 1**, we found that the high level of expression group had a higher positive rate of lymph node metastasis (*p*=0.031); however, there was no statistical difference at the TNM stage (*p*=0.081). We also found that higher levels of expression of *DEF6* mean higher levels of CA199 and larger tumor sizes. This suggests that *DEF6* may be associated with tumor development.

**Table 1:** Clinical information.

	Low IHC score (*n*=10)	High IHC score (*n*=10)	*p*-value
Age	59.6±7.8	61.7±7.4	0.544
BMI (kg/m^2^)	21.3±3.0	22.7±3.0	0.338
T stage			0.388
T0, T1, and T2	50%	30%	
T3 and T4	50%	70%	
N stage			0.031
N0	70%	30%	
N1	30%	50%	
N2+	0	20%	
TNM stage			0.081
I and II	70%	30%	
III	30%	70%	
CEA	3.5±1.5	5.2±3.9	0.223
CA199	16.7±9.9	30.2±11.8	0.012
Tumor size	2.96±1.19	4.50±1.48	0.020

### Tumor heterogeneity analysis

**Figure 4:**
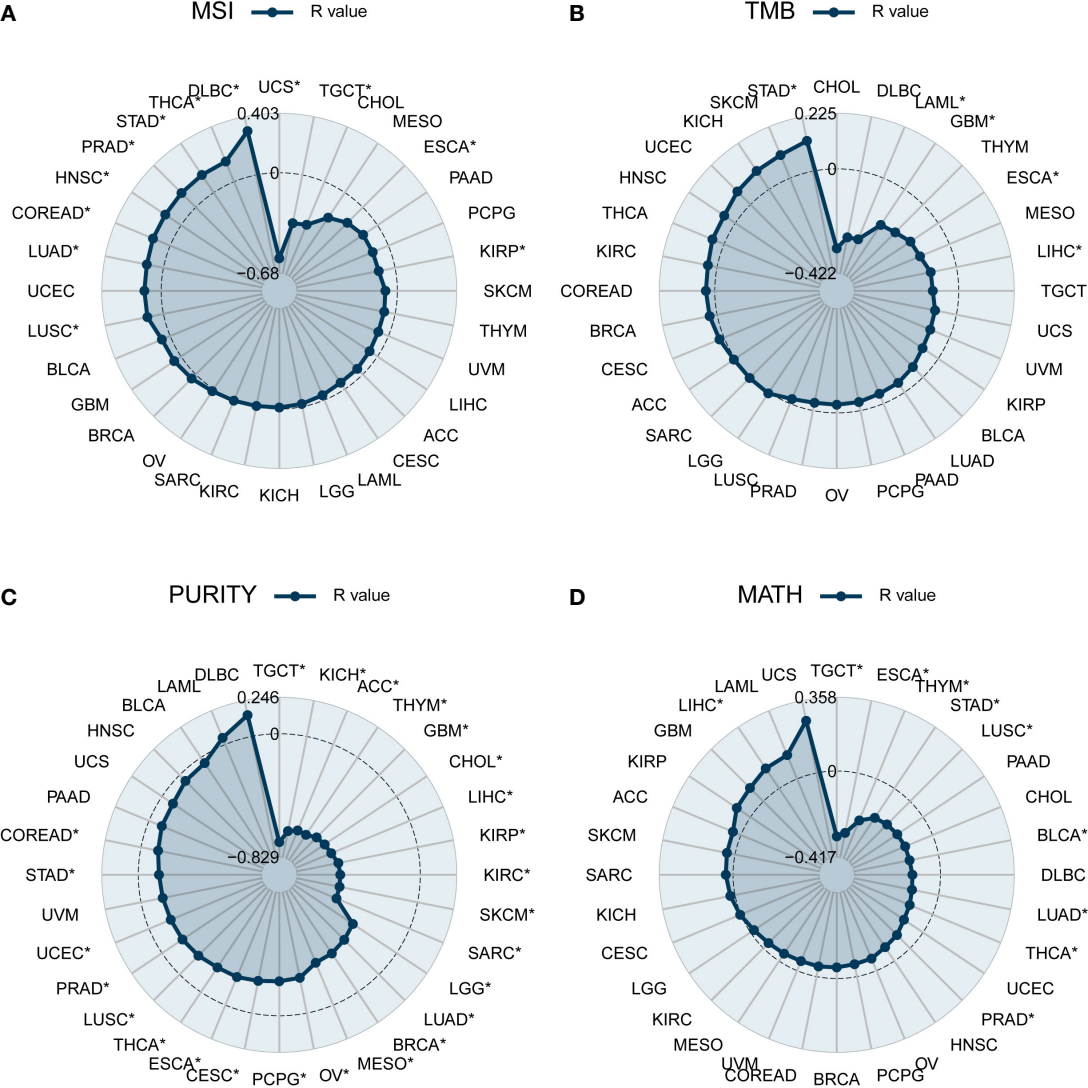
Correlation of *DEF6* expression with tumor heterogeneity. Radar plot of the correlation between *DEF6* expression and **(A)** MSI, **(B)** TMB, **(C)** tumor purity, and **(D)** MATH. MSI, microsatellite instability; TMB, tumor mutational burden; MATH, mutant-allele tumor heterogeneity. *p < 0.05. *p*-values are adjusted using the BH method.

Indicators associated with tumor heterogeneity include MSI, TMB, purity, and MATH. We calculated the correlation between these indicators and *DEF6* expression to reveal the possible effects of *DEF6* on tumors. Microsatellite instability (MSI) is caused by defects in the mismatch repair system, resulting in hypermutation patterns. The correlation between *DEF6* and MSI is demonstrated in **Figure 4A**. *DEF6* expression was significantly negatively correlated with MSI in USC, TGCT, ESCA, and KIRP, but positively correlated with MSI in LUSC, LUAD, COREAD, HNSC, PRAD, STAD, THCA, and DLBC (*p* < 0.05). As shown in **Figure 4B**, we found that the association between *DEF6* expression and TMB was not strong, showing negative correlations in LAML, GBM, ESCA, and LIHC, and positive correlations only in STAD (*p* < 0.05). **Figure 4C** demonstrates the association between *DEF6* expression and tumor purity. Tumor purity was associated with immune infiltration, and we found that tumor purity decreased significantly with increased expression of *DEF6*, which partly proves that *DEF6* expression is associated with immune infiltration. MATH is an algorithm to calculate tumor intratumor genetic heterogeneity, and greater heterogeneity is associated with a worse prognosis (30, 31). We evaluated the correlation between *DEF6* expression and MATH in **Figure 4D**. In TGCT, ESCA, THYM, STAD, BLCA, LUAD, THCA, PRAD, and LUSC, higher levels of expression of *DEF6* correlated with low MATH scores, whereas, in LIHC, MATH scores increased with higher levels of *DEF6* expression.

### Analysis of gene mutations and modifications

Gene modifications play a critical role in cancer development, and we evaluated the correlation between *DEF6* expression and methylation-modified genes, including m6A, m5C, and m1A.

**Figure 5:**
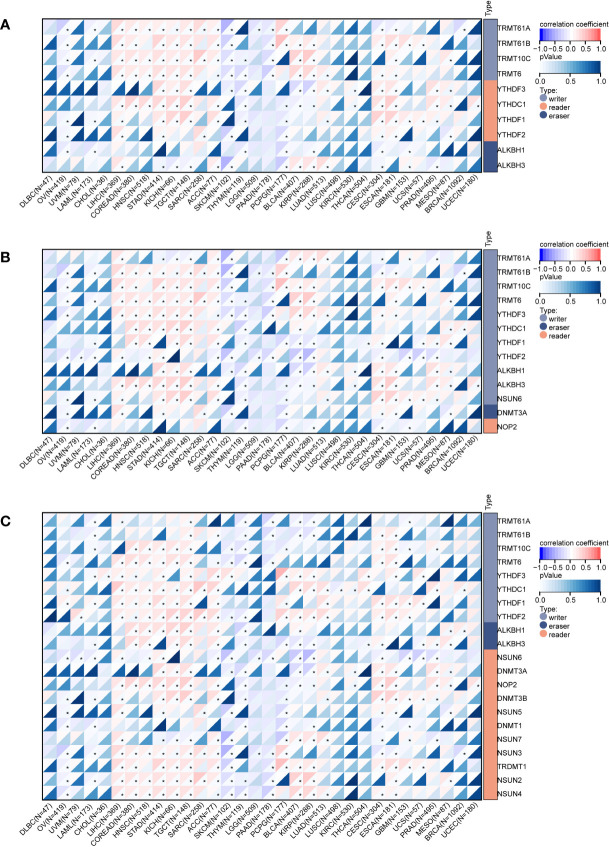
Correlation of *DEF6* expression with RNA-modified genes. Correlation of *DEF6* expression with **(A)** m1A-, **(B)** m5C-, and **(C)** m6A-modified gene expression in multiple cancers.

**Figure 5A** shows the correlation heatmap between m1A-modified genes and *DEF6* expression, and we can conclude that *DEF6* expression showed a positive correlation with m1A-modified genes in LIHC, COREAD, HNSC, STAD, KICH, TCGT, ACC, CESC, and ESCA, and a negative correlation with m1A-modified genes in OV, SKCM, THYM, and PCPG. The correlation between m5C- and m6A-modified genes and *DEF6* expression was similar to the correlation between *DEF6* expression and m1A-modified genes, being mainly positive in LIHC, COREAD, HNSC, STAD, KICH, TGCT, and ACC, and negative in SKCM and THYM (**Figures 5B, C**).

**Figure 6:**
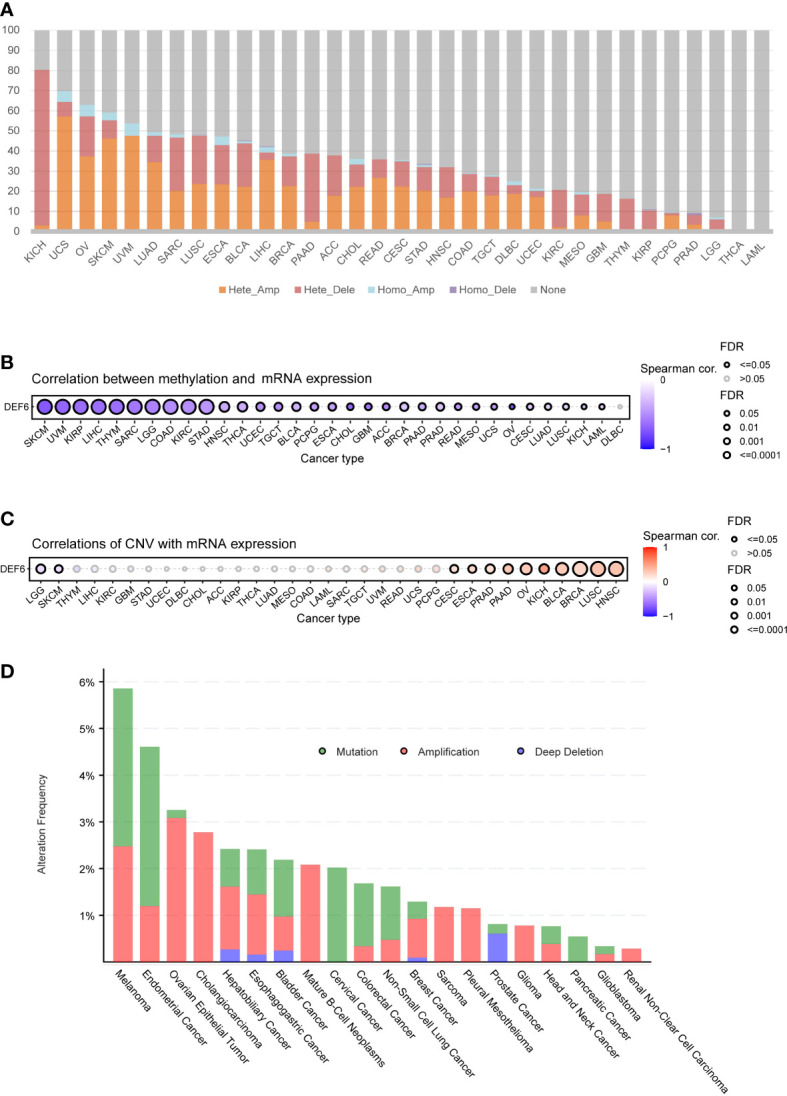
*DEF6* and genetic alteration profiles. **(A)** The bar plot summarizes the CNV of *DEF6* in a variety of cancers. **(B, C)** The correlation between methylation and CNV with *DEF6* mRNA expression. **(D)** *DEF6* alteration frequencies in various cancers. *p*-values are adjusted by the FDR method. CNV, copy number variation.

We analyzed the association between *DEF6* expression and gene mutations. First, we obtained the mutation data and extracted the copy number variation data of *DEF6*. We found that *DEF6* expression was predominantly associated with heterozygous deletion in KICH, with a very high mutation rate (77.27%), whereas in SARC (26.46%), LUSC (23.95%), BLCA (21.57%), PAAD (33.70%), and KIRC (18.56%), heterozygous deletion also accounted for the majority of mutation types. In other cancers, heterozygous amplification was the predominant form of mutation; interestingly, *DEF6* mutations were not found in THCA and LAML (**Figure 6A**). Previously, we analyzed the correlation between RNA-modified genes and *DEF6* expression; subsequently, we analyzed the correlation between *DEF6* expression and the degree of methylation. Surprisingly, as can be seen in **Figure 6B**, *DEF6* expression showed a significant negative correlation with methylation. By contrast, the correlation between *DEF6* expression and copy number variation was mainly positive in HNSC, LUSC, BRCA, BLCA, KICH, OV, PAAD, PRAD, ESCA, and CESC, but negative in LGG and SKCM (**Figure 6C**). We explored the genetic alterations of *DEF6* in the TCGA pan-cancer datasets through the cBioPortal online resource. The results revealed that the overall frequency of *DEF6* was relatively high in cancers and dominated by “copy number mutations”, with the highest value being found in melanoma, at over 5% (**Figure 6D**).

### Immunological genes and DEF6

**Figure 7:**
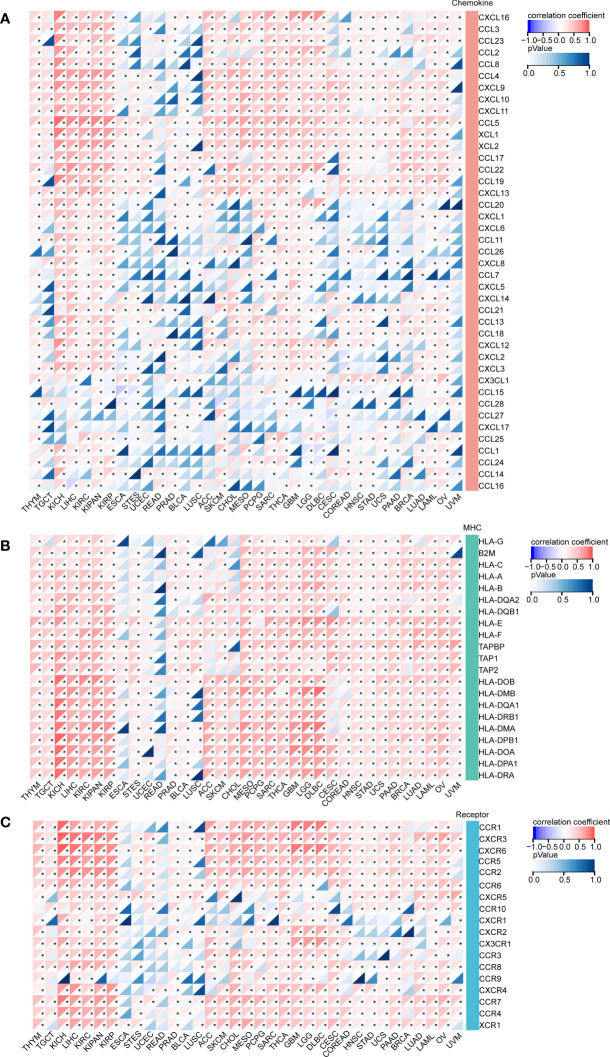
The correlation between *DEF6* expression and immune related genes. **(A)** The heat map demonstrates the correlation of *DEF6* expression with **(A)** chemokines, **(B)** MHC, and **(C)** receptor genes in a variety of cancers. MHC, major histocompatibility complex. *Correlation *p* < 0.05. *p*-values are adjusted using the BH method.

Since *DEF6* deficiency is linked to several immunological disorders, we surmised that *DEF6* may also be relevant to genes that regulate the immune system in cancer. Therefore, we analyzed the correlation between *DEF6* expression and chemokine, MHC, and receptor genes. As shown in **Figure 7A**, *DEF6* expression showed a strong positive correlation with chemokines by correlation analysis in TGCT, KICH, LIHC, KIRC, KIRP, ACC, SKCM, CHOL, MESO, PCPG, SARC, THCA, GBM, and LGG. CCL5, XCL1, XCL2, CCL17, CCL22, and CCL19 showed a positive correlation with *DEF6* in most cancers, and *DEF6* may function in regulating these chemokines in cancer. The main functions of the MHC include participation in antigen presentation and processing, which plays an important function in cancer immunity. The results, as shown in **Figure 7B**, indicate that there was a significant positive correlation between *DEF6* expression and MHC regulatory genes. With the exception of ESCA, UCEC, READ, and LUSC, there was a general trend toward a larger number receptor-regulated genes as *DEF6* expression increased (**Figure 7C**).

### Tumor microenvironment and immune cell infiltration analysis

**Figure 8:**
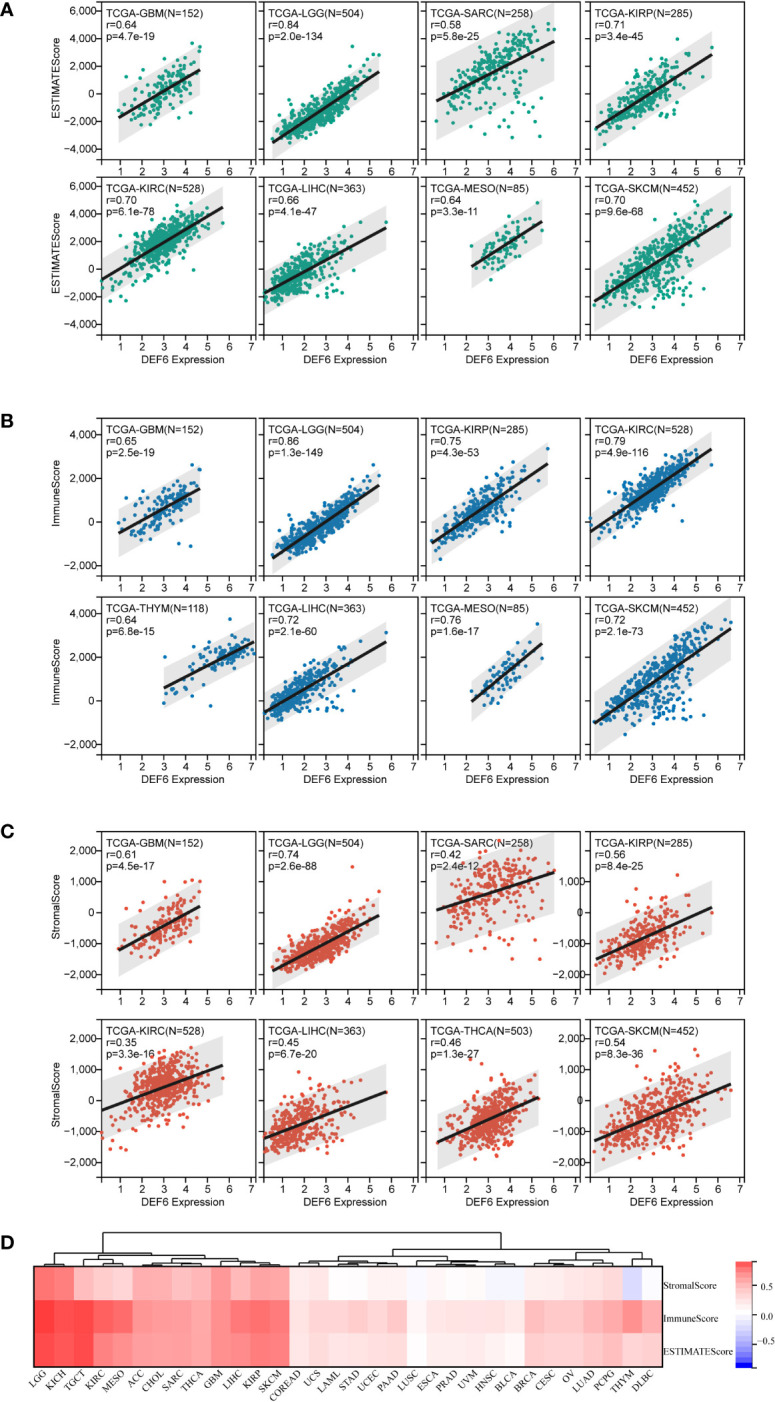
Correlation of *DEF6* expression with immune infiltration. **(A–C)** The eight types of cancer with the strongest correlation between *DEF6* expression and ESTIMATE score, IMMUNE score, and stromal score. **(D)** The heatmap demonstrates the overall profile of the correlation between *DEF6* expression and immune infiltration scores.

Malignant tumor tissues include not only tumor cells, but also normal epithelial and stromal cells, immune cells, and vascular cells associated with the tumor. Stromal cells are closely associated with tumor growth, disease progression, and tumor resistance. We used the ESTIMATE algorithm, including stromal score, immune score, and ESTIMATE score, to estimate the relationship between *SUSD4* expression level and tumor microenvironment (TME) (**Figure 8**). **Figure 8A–C** shows the top eight cancers with the strongest correlation between *DEF6* expression and ESTIMATE score, immune score, and stromal score. We discovered that the three immune infiltration scores significantly increased in GBM, LGG, SARC, KIRP, KIRC, LIHC, and KICH with an increase in level of expression of *DEF6*, suggesting that *DEF6* expression may be crucial for immune infiltration in these malignancies. Finally, **Figure 8D** provides an overview of the correlation between *DEF6* expression and immune infiltration in 32 cancers.

### Enrichment analysis

**Figure 9:**
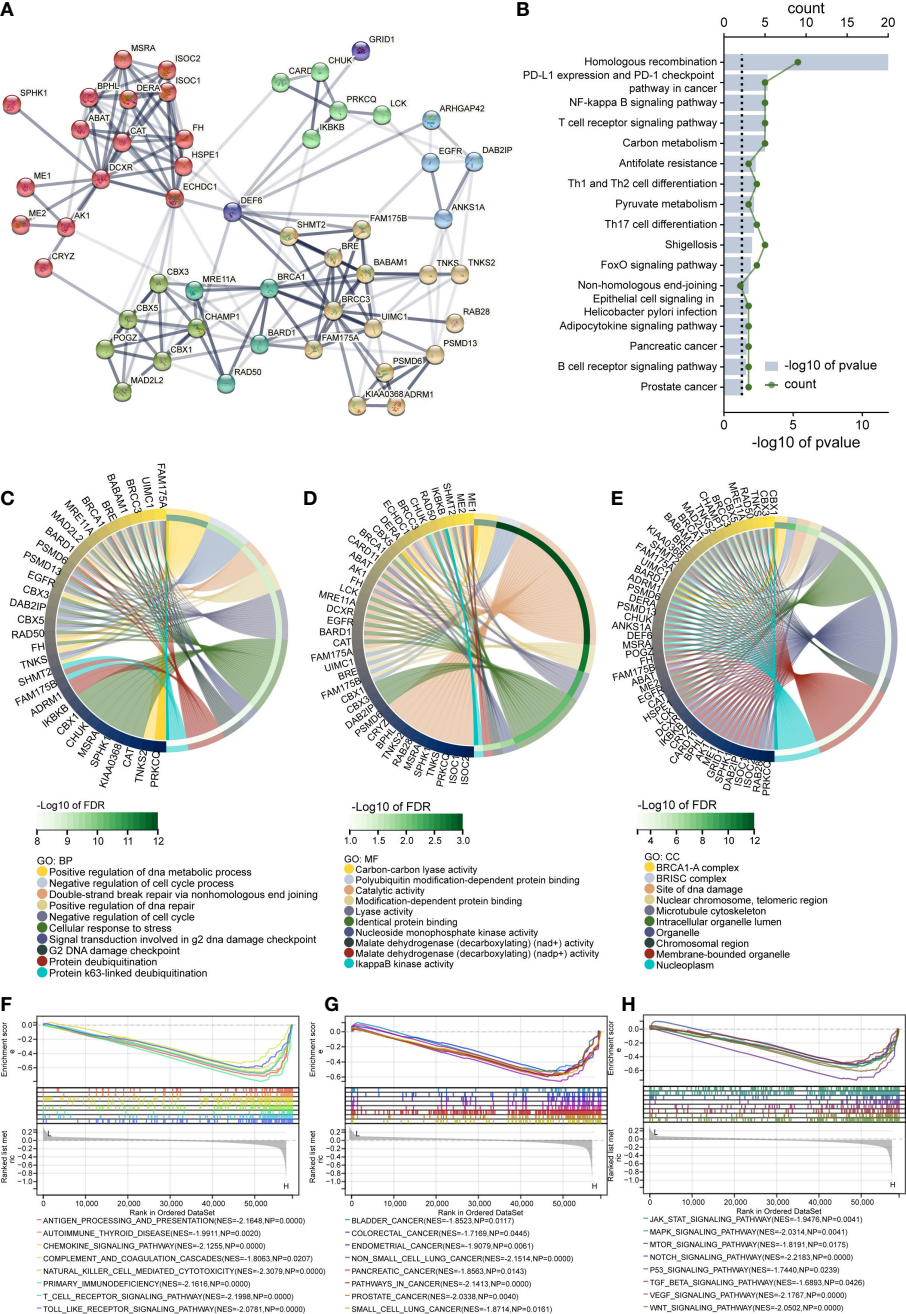
The results of *DEF6* enrichment analysis. **(A)** Protein–protein network of *DEF6* generated by the STRING database. **(B)** Top terms of KEGG enrichment analysis of protein–protein interaction networks. The chord diagram shows the **(C)** BP, **(D)** MF, and **(E)** CC results of GO enrichment analysis. **(E–G)** Results of enrichment analysis of GSEA in colorectal cancer. CC, cellular component; BP, biological process; MF, molecular function; GO, gene ontology. KEGG, Kyoto encyclopedia of genes and genomes. *p*-values are adjusted using the FDR method in GO and KEGG analysis.

Previous findings suggest that *DEF6* expression may play a key role in cancer, and using enrichment analysis we expect to elucidate the pathways and activities. First, we performed the analysis of the PPI network with *DEF6* by STRING in **Figure 9A**. Using genes related to *DEF6* as input for KEGG enrichment analysis, we found that, consistent with predictions, multiple immune-related pathways could be enriched (**Figure 9B**).

**Figures 9C, D** shows the top 10 terms of GO enriched by PPI genes. We found that *DEF6* expression may play a function in regulating the cell cycle, and, as shown in **Figure 9C**, we enriched a variety of cell cycle-related functions. In COREAD, the level of *DEF6* expression was high in cancerous tissues, and a high level of expression was associated with worse prognosis. We grouped *DEF6* according to median *DEF6* expression and performed GSEA, and the results are shown in **Figure 9E–G**. We enriched many immune-related pathways, as shown in **Figure 9E**. *DEF6* expression may be involved in the tumor immune process, affecting the development and progression of COREAD through chemokines, immune cell infiltration, etc. **Figure 9F** shows the enriched cancer-related pathways, and these results suggest that *DEF6* expression may indeed be associated with cancer development. We found that the MAPK pathway, the TGF-β pathway, the WNT pathway, and the VEGF pathway were significantly enriched. These pathways are closely related to cancer, and *DEF6* expression may interact with or regulate these pathways (**Figure 9G**).

## Discussion

*DEF6* is a gene associated with human immunity, and its deficiency is closely related to autoimmune diseases (5, 6). There are only a few studies on the relationship between *DEF6* expression and cancer, and it is believed that *DEF6* expression contributes to cancer initiation and worse prognosis. It is necessary to explore the role of *DEF6* expression in cancer through big data as well as bioinformatics. As the association of *DEF6* expression with cancer has been reported in only a few tumors, we performed an expression versus survival differential analysis. Using the TCGA and GTEx databases, we found aberrant expression profiles of *DEF6* in cancer. Consistent with previous reports, levels of *DEF6* expression were highest in immune cells in the expression analysis at the cell line level. We found that in ACC, LUAD, PRAD, SLCM, STAD, TGCT, and THCA, *DEF6* expression was significantly lower in cancer tissues. Atypically high levels of *DEF6* expression may be a sign of carcinogenesis or tumor development in various malignancies. By analyzing patient survival data, we discovered that levels of *DEF6* expression in COREAD were abnormally high and that patients with high levels of expression had considerably worse prognoses, indicating that *DEF6* expression may have a unique role in promoting cancer in this disease. Coincidentally, prognoses were better in SKCM and LUAD, where there were high levels of expression of *DEF6*. This may indicate that *DEF6* exhibits pleiotropy in different cancers, rather than acting only as an oncogene. We speculate that the function of *DEF6* varies in different tumors. *DEF6* acts as a carcinogenic gene in some tumors, and has a protective effect in others. We believe that this phenomenon deserves to be studied in depth, but it requires the involvement of experts from a wider range of fields.

We found that patients with higher IHC scores had more lymph node metastases, higher levels of CA199, and larger tumors. This further validates our hypothesis. These results suggest that *DEF6* contributes to the promotion of lymph node metastatic function in colorectal cancer. We speculate that tumor cells with high levels of *DEF6* expression may have greater metastatic capacity, but this needs to be verified *in vitro*. Ki67 is widely used in pathological diagnosis, and it is generally accepted that Ki67 is closely associated with tumor metastasis and stage (32). In colorectal cancer, immunohistochemistry for *DEF6* can be used for lymph node staging and may be adopted in clinicopathological diagnosis after more in-depth validation.

DNA methylation is a key epigenetic process that is critical for the regulation of gene expression. There is much evidence that aberrant DNA methylation is associated with tumorigenesis and cellular aging, and we assessed the relationship between *DEF6* expression and DNA methylation (33, 34). Consistent with expectations, *DEF6* expression showed a significant negative correlation with DNA methylation in the majority of tumors.

Further, we calculated the correlation of *DEF6* expression with multiple indicators of tumor heterogeneity. The results showed us a significant correlation between the expression of *DEF6* and MSI and tumor purity. The association between tumor purity and immune cell infiltration may indicate that *DEF6* is involved in immune recruitment, although this was not confirmed in the current study, necessitating further investigation by other researchers. Previous studies have reported that *DEF6* defects might cause immune diseases, and we speculate that mutations in *DEF6* might also be present in tumors (6, 35). Consistent with expectations, the frequency of mutations in *DEF6* in multiple cancers exceeded 50%. Perhaps the *DEF6* mutation is also responsible for the development of cancer.

The known clues about *DEF6* all point to immunity; therefore, we investigated the correlation between *DEF6* expression and multiple immune genes. The results showed a close relationship between *DEF6* expression and tumor immune genes and a robust correlation between *DEF6* expression and immune infiltration score. Immune cells in the TME play an essential role in tumorigenesis and may act to promote the growth of tumors (36). As a result of cross-talk between cancer cells and immune cells, an environment is created that favors tumor growth and metastasis, which have a robust correlation between *DEF6* expression in tumors (37). *DEF6* has the potential to be an immunotherapeutic target.

*DEF6* could have more than just immune-related functions; other mechanisms are unknown, and we speculate on the possible functions of *DEF6* through enrichment analysis. As expected, the pathways relevant for *DEF6* enrichment include immune cells and immunomodulatory pathways. The PD1 and PDL1 pathways are also included the enrichment results; perhaps the expression of *DEF6* can guide the application of PD1 treatment, but this should be verified by more *in vivo* experiments. Other enrichment analyses suggest that *DEF6* may also be involved in purine metabolism, carbon metabolism, and multiple cancer pathways. *DEF6* not only is abnormally expressed in colorectal cancer but is also closely related to prognosis. We performed GSEA in the colorectal cancer dataset, hoping to find the possible functions of *DEF6*. *DEF6* may interact with various types of cancer-related signaling, including the JAK/STAT pathway, MAPK pathway, NOTCH pathway, and VEGF pathway in colorectal cancer.

There are some limitations to this study; firstly, there is some bias in the bioinformatic analysis, which may lead to unreliable results, and, secondly, this study was not validated *in vivo* and *in vitro*. We need to perform *in vivo* or *in vitro* studies to complement the validation of the role and function of *DEF6*. Finally, although we used colorectal cancer samples for the analysis of immunohistochemistry with clinical information, other cancers were not validated. Overall, this study provides a comprehensive pan-cancer analysis for *DEF6*, although there are some limitations.

## Data availability statement

The original contributions presented in the study are included in the article/**Supplementary Material**. Further inquiries can be directed to the corresponding author.

## Ethics statement

Ethics approval for this study was obtained from the Second Affiliated Hospital of Harbin Medical University. Written informed consent for participation was not required for this study in accordance with the national legislation and the institutional requirements.

## Author contributions

ZY conceived the research, analyzed the data, and drafted the work. YZ analyzed the data and performed the visualization. HH and WZ collected the data and participated in the revision. GW supervised the study. All authors contributed to the article and approved the submitted version.

## Supplementary material

Supplementary Figure 1 Immunohistochemistry of colorectal cancer and paired samples. **(A)** Immunohistochemistry of colorectal cancer tissue. **(B)** Immunohistochemistry of paired normal colorectal tissue. **(C)** IHC scores for cancerous versus normal tissue. *****p* < 0.0001.
