# Supplementary figures and images for: DEF6 has potential to be a biomarker for cancer prognosis: A pan-cancer analysis
Source: Front Oncol. 2023 Jan 4;12:1064376. doi: 10.3389/fonc.2022.1064376 (PMC9848736; doi:10.3389/fonc.2022.1064376)

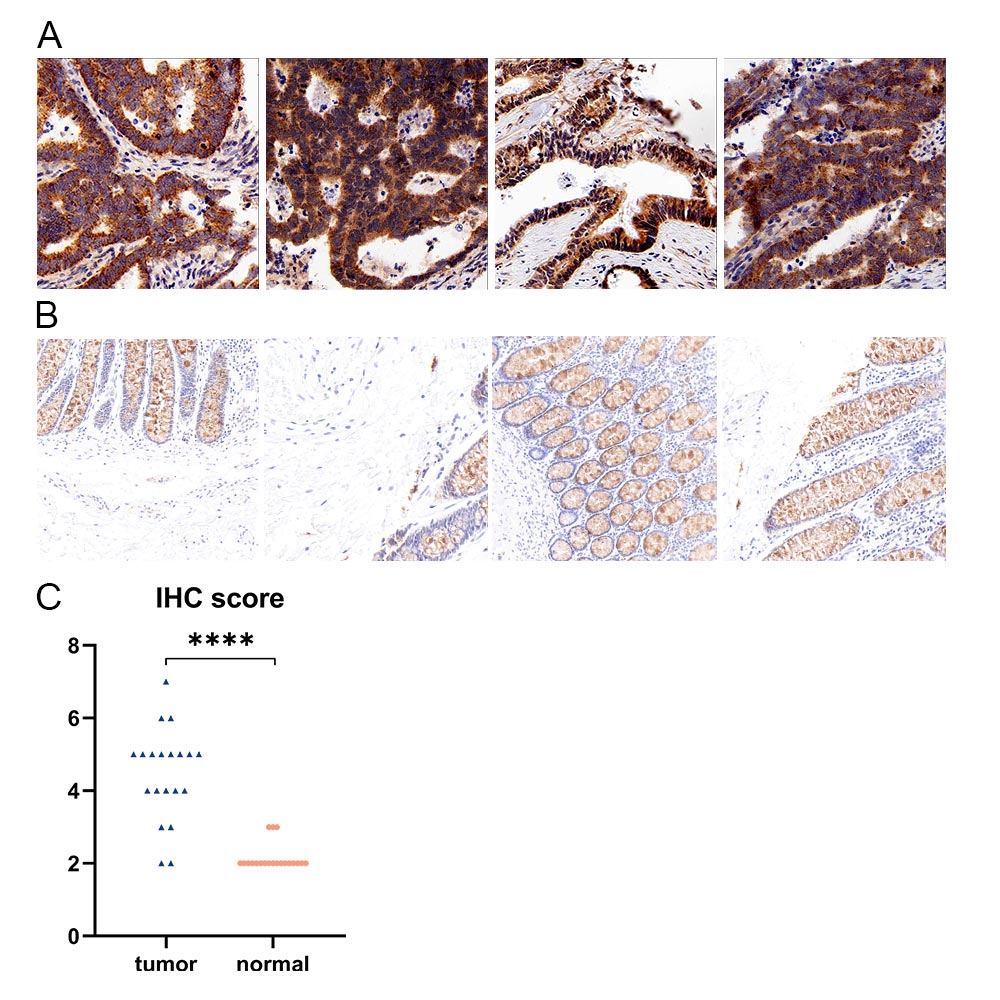

Supplement: Supplementary Figure 1 — Immunohistochemistry of colorectal cancer and paired samples. (A) Immunohistochemistry of colorectal cancer tissue. (B) Immunohistochemistry of paired normal colorectal tissue. (C) IHC scores for cancerous versus normal tissue. ****p < 0.0001. [file Image_1.jpeg]
